# Diagnostic Performance of Procalcitonin for the Early Identification of Sepsis in Patients with Elevated qSOFA Score at Emergency Admission

**DOI:** 10.3390/jcm10173869

**Published:** 2021-08-28

**Authors:** Myrto Bolanaki, Martin Möckel, Johannes Winning, Michael Bauer, Konrad Reinhart, Angelika Stacke, Peter Hajdu, Anna Slagman

**Affiliations:** 1 Department of Emergency and Acute Medicine, Campus Virchow and Mitte, Charité—Universitätsmedizin Berlin, Augustenburger Platz 1, 13353 Berlin, Germany; martin.moeckel@charite.de (M.M.); peter.hajdu@charite.de (P.H.); anna.slagman@charite.de (A.S.); 2 Klinik für Anästhesiologie und Intensivmedizin, Uniklinikum Jena, Am Klinikum 1, 07747 Jena, Germany; Johannes.Winning@med.uni-jena.de (J.W.); Michael.Bauer@med.uni-jena.de (M.B.); 3 Klinik für Anästhesiologie m. S. Operative Intensivmedizin, Charité—Universitätsmedizin Berlin, Augustenburger Platz 1, 13353 Berlin, Germany; Konrad.Reinhart@charite.de; 4 Ernst-Abbe-Hochschule, University of Applied Sciences, Carl-Zeiss-Promenade 2, 07745 Jena, Germany; angelika.stacke@med.uni-jena.de

**Keywords:** qSOFA, SOFA, sepsis, procalcitonin

## Abstract

Infectious biomarkers such as procalcitonin (PCT) can help overcome the lack of sensitivity of the quick Sequential Organ Failure Assessment (qSOFA) score for early identification of sepsis in emergency departments (EDs) and thus might be beneficial as point-of-care biomarkers in EDs. Our primary aim was to investigate the diagnostic performance of PCT for the early identification of septic patients and patients likely to develop sepsis within 96 h of admission to an ED among a prospectively selected patient population with elevated qSOFA score. In a large multi-centre prospective cohort study, we included all adult patients (*n* = 742) with a qSOFA score of at least 1 who presented to the ED. PCT levels were measured upon admission. Of the study population 27.3% (*n* = 202) were diagnosed with sepsis within the first 96 h. The area under the curve for PCT for the identification of septic patients in EDs was 0.86 (95% confidence interval (CI): 0.83–0.89). The resultant sensitivity for PCT at a cut-off of 0.5 µg/L was 63.4% (95% CI: 56.3–70.0). Furthermore, specificity was 89.2% (95% CI: 86.3–91.7), the positive predictive value was 68.8% (95% CI: 62.9–74.2), and the negative predictive value was 86.7% (95% CI: 84.4–88.7). The early measurement of PCT in a patient population with elevated qSOFA score served as an effective tool for the early identification of sepsis in ED patients.

## 1. Introduction

### 1.1. Background

In 2016 the Third International Consensus Definitions for Sepsis and Septic Shock redefined sepsis as a life-threatening organ dysfunction resulting from a dysregulated host response to an infection [1]. Organ dysfunction was defined as an acute increase of at least 2 points in the patient’s Sequential Organ Failure Assessment (SOFA) score, a well-known measure first introduced in 1994 [2] as a tracking tool for organ failure in intensive care units (ICUs). The worldwide incidence of sepsis is estimated to be approximately 48.9 million cases per year [3,4], with persistently high morbidity and mortality rates [5]. This high incidence of sepsis coupled with poor clinical outcome places a heavy burden on health care systems and consumes a high proportion of already scarce hospital resources (e.g., personnel, hospital beds, intensive care capacity). According to a recent study by Buchman et al. conducted in the USA, the human and economic costs of sepsis care continue to grow [6,7,8].

To improve sepsis outcomes and avoid excessive resource consumption due to extended but preventable in-hospital treatment and intensive care stays, early identification of septic patients and patients likely to develop sepsis is a top clinical priority to enable early strategy implementation. According to the latest update to the surviving sepsis campaign guidelines, early management should involve early fluid resuscitation, lactate measurement, obtaining of blood cultures, early administration of broad-spectrum antibiotics, and, where necessary, the application of vasopressors within one hour of diagnosis [9].

In order to achieve early sepsis recognition, the Third International Consensus Definitions for Sepsis and Septic Shock (Sepsis-3) searched for the most suitable criteria for the development of an effective sepsis-screening tool. Consequently, the quick SOFA (qSOFA) score was introduced. This score consists of three easy-to-assess parameters: SBP ≤ 100 mmHg, respiratory rate ≥ 22 breaths per minute, and altered cognitive state. To define altered cognitive state the use of the Glasgow Coma Scale (GCS) was recommended. This may have limited the detection of patients at risk, since the GCS was intended to describe the extent of impaired consciousness and not altered mentation [10]. Seymour et al. [11] proved the qSOFA score’s prognostic superiority to the previously used systemic inflammatory response syndrome (SIRS) criteria for non-ICU patients with suspected infection and underlined its ideal use outside ICUs and in emergency departments. According to the 2018 Surviving Sepsis Campaign (SSC) guidelines for the management of sepsis and septic shock, a qSOFA score of 2 or higher (i.e., a positive qSOFA score) should prompt physicians to look for evidence of organ dysfunction and to search for sepsis in order to improve the patient’s outcome. However, the absence of a positive qSOFA score should not be misinterpreted as the absence of sepsis. The Sepsis-3 task force suggested that further research should be conducted to evaluate the prognostic and diagnostic performance of the score and proposed using the qSOFA criteria as entry criteria in clinical trials in the future.

After the introduction of the qSOFA score, numerous studies tested the prognostic performance of the score in variant study populations; the results were conflicting. Specifically, studies involving ED patients with suspected or confirmed infection exhibited relatively low sensitivity for the qSOFA score in predicting in-hospital mortality [12,13,14]. Similarly disappointing results were published regarding the sensitivity of the score for sepsis diagnosis [15,16]; however, sensitivity increased slightly when a qSOFA score cut-off of 1 was applied [16].

Because of these results, improving the prognostic performance of the qSOFA score became the primary goal of research. Subsequent studies [17,18,19] revealed that the addition of multiple infectious disease biomarkers—including procalcitonin (PCT), mid-regional proadrenomedullin, and multiple interleukins—could counterbalance the absence of a parameter that reflects infection in the qSOFA score and therefore improve its prognostic accuracy. Procalcitonin, a member of the calcitonin family, is a biomarker that rises rapidly during an infection and has already been established to monitor response to and guide antimicrobial therapy in septic patients [20,21]. Furthermore, PCT has been proved to be an important biomarker for sepsis diagnosis and prognosis as summarised in several systematic reviews [22]. Additionally, several studies [23,24] also showed that procalcitonin could be used to differentiate infectious and non-infectious inflammatory diseases, making it the ideal additional biomarker for a qSOFA-elevated population.

To date, only two studies have evaluated the diagnostic performance of PCT combined with qSOFA [25,26]. The previously mentioned studies provided encouraging results; however, they were not conducted in ED settings and did not apply the qSOFA criteria as inclusion criteria to select their study populations. They rather analysed patients who had been identified as having suspected infection or had been retrospectively diagnosed with sepsis.

### 1.2. Research Aim

The aim of the current study was to determine the diagnostic performance of PCT for early sepsis recognition in a population of ED patients with a qSOFA score of at least 1 upon admission. PCT was additionally investigated as a potential predictor of 30-day mortality in patients with elevated qSOFA scores in the ED.

## 2. Materials and Methods

### 2.1. Study Design

The LIFE-POC study was a large multi-centre prospective cohort study conducted in three EDs at three tertiary care hospitals: Charité University Hospital Berlin (Campus Virchow Klinikum and Campus Charité Mitte) and Jena University Hospital. The analysis included all adult patients (*n* = 742) admitted between 1 January 2017 and 23 March 2018 at the Berlin study sites with a qSOFA score of at least 1.

This study was approved by the institutional review boards of the respective universities. Written informed consent was obtained from all patients or their legal representatives where appropriate.

### 2.2. Patient Selection

All patients aged ≥ 18 years who had a qSOFA score of at least 1 were included in the study. Patient recruitment was conducted for seven days a week on a daily 8-h basis in alternating day and night shifts. The qSOFA score of each patient who presented at the ED during the study period was assessed, regardless of suspicion of infection, and confirmed by the study team at enrolment. The inclusion of patients with one qSOFA point was chosen to also detect patients likely to develop sepsis in the ED.

Patients suffering from acute trauma, an acute ST-elevation myocardial infarction, or suspected stroke or had been admitted for palliative care with a life expectancy of less than 1 month were excluded from the study. Further exclusion criteria involved pregnancy and referrals from other hospitals following prior in-hospital treatment. Additionally, patients were only included once in the study. All the study patients were treated according to the standard best practices of the department in question and current clinical guidelines.

### 2.3. Blood Sampling and Biomarker Measurement

Blood samples were collected within 12 h of presentation at the ED following written informed consent. For each patient, a sample of 18 mL of whole blood was taken after venipuncture under aseptic conditions, divided into three sampling tubes (ethylenediaminetetraacetic acid (EDTA), lithium heparin, and serum separator tubes) and centrifuged at room temperature. Plasma was immediately aliquoted, and the aliquots of all materials were stored at −20 °C within four hours. Blood samples were shipped on dry ice to the research laboratory at Charité University Hospital Berlin on the same day and stored at −80 °C within 72 h. Once a week, all the aliquots were shipped to the central biobank at the Jena University Hospital, where they were stored at −80 °C until measurement.

PCT was measured in all the study patients with an automated immunofluorescent assay using a Brahms PCT sensitive Kryptor. The direct measurement range of the assay was 0.02 to 50 µg/L, and the measurement range with automatic dilution was 0.02 to 5000 µg/L. The 95th percentile of serum or plasma PCT concentrations in healthy persons with this assay was 0.064 µg/L. The detection limit, calculated using the imprecision profile, was 0.02 µg/L. The following cut-offs for PCT [27,28] were investigated in the current analysis: 0.25, 0.5, 2.0, 5.0, and 10 µg/L.

### 2.4. Data Collection

Primary data were obtained in the EDs, starting with screening at triage. Clinical routine data were then extracted from the clinical patient records system. All data were then entered into a study-specific electronic case report form by members of the study personnel. The clinical study data included the patient’s medical history, admission data, process data, clinical in-hospital courses (including medications), vital signs, laboratory findings, and diagnostic procedures. In addition, 28-day follow-up calls were arranged to assess further clinical courses and 28-day mortality. The study database was coordinated by the clinical study centre of the University of Jena. Data were monitored and checked for plausibility by data managers on a regular basis.

### 2.5. Endpoints

The patients’ electronic or paper medical files were consistently reviewed for 4 calendar days or until discharge from the hospital. The primary endpoint of this study was the diagnosis of sepsis within 96 h. Our aim was to include all patients with community-acquired sepsis, since these are the most common ones [29,30] and usually present to EDs in need of early sepsis management [31]. Furthermore, the board of the clinical trial aimed to include late sepsis development so that early biomarker diagnostic performance could be assessed. Direct referrals from other clinics were excluded.

In accordance with the Sepsis-3 criteria, sepsis was diagnosed as an acute change in a patient’s total SOFA score of ≥2 points due to infection. Therefore, each patient’s SOFA score was calculated daily for the first 4 days. Sepsis diagnoses were adjudicated by an expert panel. PCT values, if measured in clinical routine, were available for the expert panel to examine. However, the PCT values measured as part of the study were not available. Non-septic patients served as the control group for this study. The secondary study endpoint was 28-day mortality, which was defined as all-cause mortality within 28 days of initial enrolment.

### 2.6. Statistical Analysis

The study data were analysed in SPSS version 25 (IBM Deutschland GmbH, Ehningen, Germany). The intended sample size was 750 patients, as determined based on feasibility considerations. Relative and absolute frequencies were reported, and the chi-squared test was conducted for statistical comparisons among two or more groups in terms of categorical variables. The distribution of numeric variables was investigated graphically using histograms, normal distribution approximation curves, and the Kolmogorov–Smirnov test. Owing to skewed distributions of numeric variables, median values and interquartile ranges (IQRs) were reported, and non-parametric statistical tests (Mann–Whitney U Test) were conducted. A *p*-value below 0.05 was considered statistically significant. The diagnostic utility of biomarkers was primarily assessed and graphically illustrated by the area under the receiver operating characteristic curve (AUROC). Diagnostic performance was quantified based on sensitivity, specificity, positive predictive value (PPV), negative predictive value (NPV), and accuracy and was calculated from crosstabs for the diagnosis of sepsis within the first 96 h (primary endpoint). Logistic regression analyses were performed for the primary endpoint as the dependent variable, with PCT as a binary variable at the aforementioned cut-off points. Odds ratios (ORs) and in addition, 95% confidence intervals (CIs) were reported. Logistic regression was conducted for PCT as a single predictor (crude OR), adjusted for qSOFA (model 1); qSOFA, sex, and age (model 2; age entered as a numeric variable); and qSOFA, sex, age, C-reactive protein (CRP), and lactate (model 3; age and other biomarkers entered as numeric variables).

In the classification tree analysis, sepsis within 96 h was entered as the dependent variable, and the independent variables were (1) PCT with a fixed number of four group intervals and (2) the qSOFA score for all three categories. The minimum group sizes were set as 70 for the superior nodes and 10 for the inferior nodes. The chi-square automatic interaction detection (CHAID) method was used, allowing for a maximum of five steps, and the significance level for both nodes was split, and node consolidation was 0.05. Significance correction was conducted using the Bonferroni method. The model estimation criteria allowed for a maximum number of 100 iterations and a minimum change in the expected cell frequencies of 0.001. For validation purposes, a training and test data set was randomly sampled from the study population, and the algorithm derived from the training data set was then applied in the test (validation) data set.

The net reclassification improvement (NRI) was calculated using established risk cut-off values for qSOFA (2 points) and PCT 0.50 µg/L and again applying optimised cut-off values, which were identified in classification tree analysis (PCT: 0.13 µg/L and 0.50 µg/L).

## 3. Results

### 3.1. Patient Population

All patients who presented at one of the two EDs in Berlin during the study period were screened, and 742 patients who met the inclusion criteria and agreed to participate in our study were included (Supplementary Figure S1, CONSORT flow diagram). Of these 742 patients, 42.0% (*n* = 312) were women, and the median age was 68 (IQR: 56–78) years. Regarding qSOFA scores the highest proportion of patients had a score of 1 (77.1%; *n* = 572), while 20.9% (*n* = 155) had a score of 2, and 2.0% (*n* = 15) had a score of 3. The median time between presentation at the ED and study blood drawing was 2 h (IQR: 1–3 h).

Further details on clinical characteristics are provided in Table 1 and further information on comorbidities is available in Supplementary Table S1.

### 3.2. Further Clinical Course and Clinical Endpoints

After initial treatment in an ED, 24.8% (*n* = 184) of the study patients were discharged home, 44.2% (*n* = 328) were admitted to a general ward, 17.5% (*n* = 130) were admitted to an ICU, and 13.7% (*n* = 102) were transferred to another hospital.

The diagnosis of sepsis based on the Sepsis-3 definition within the first 96 h was assigned to 27.3% (*n* = 202) of the study patients. The onset of sepsis was prevalent upon admission in 20.8% (*n* = 154), occurred within the first 24 h after admission in 3.9% (*n* = 29), and occurred between 24 and 96 h after admission in 2.3% (*n* = 17) (the time of onset was unknown in two septic patients). After stratification based on qSOFA scores, it was determined that sepsis had occurred in 19.6% (*n* = 112) of all the patients with a qSOFA score of 1, 49.7% (*n* = 77) of all those with a qSOFA score of 2, and 86.7% (*n* = 13) of all those with a qSOFA score of 3.

The suspected focus of infection on day 0 was pulmonary in 38.0% (*n* = 280) of the study patients, urogenital in 8.9% (*n* = 66), and abdominal in 7.8% (*n* = 58). Less frequent infection foci were skin or wounds (3.8%; *n* = 28), other (2.2%; *n* = 16), the cardiovascular system (0.8%; *n* = 6), and the central nervous system (0.3%; *n* = 2). The infection focus was unknown in 7.4% (*n* = 55) of the patients, and no focus of infection was suspected in 31.3% (*n* = 231). The distribution of qSOFA points within infect foci is shown in Supplementary Table S2.

The distribution of infectious foci was similar in patients with sepsis within the first 96 h after admission, with 44.6% pulmonary (*n* = 90), 17.3% urogenital (*n* = 35), 11.9% abdominal (*n* = 24), 6.0% skin or wounds (*n* = 12), 3.0% cardiovascular system (*n* = 6), and 1.0% central nervous system (*n* = 2).

Microbiological tests were performed on 53.1% (*n* = 394) of the study patients within 96 h of admission. Regarding the patients who were transferred to other hospitals (*n* = 102), the study team was informed of only the microbiological tests with positive results as opposed to all such tests.

In the septic subgroup, microbiological samples were obtained from 90.6% (*n* = 183) of the study patients, and a relevant pathogen was identified in 54.7% (*n* = 110). The most common pathogens detected in the first microbiological examination within the first 96 h were Gram-negative bacteria (25.6%; *n* = 52), Gram-positive bacteria (15.9%; *n* = 32), multiple pathogens (7.0%; *n* = 14), fungal infections (4.5%; *n* = 9), other infections (1.0%; *n* = 2), and parasites (0.5%; *n* = 1).

Overall, a clinically confirmed infection was diagnosed in 28.7% (*n* = 213) of the study patients. A clinically confirmed infection was defined as an infection verified by (1) imaging features or after surgical intervention, (2) a positive urine dip test with symptoms of urinary tract infection, or (3) in cases of skin infections, typical appearance and symptoms according to a dermatologist. Suspected infection, defined as suspicion of infection according to the treating physician based on clinical signs and laboratory findings, was diagnosed in 38.4% (*n* = 285) of the study patients. The remaining patients (32.9%, *n* = 244) did not have a clinically confirmed infection or suspicion of an infection.

In the sepsis subgroup, a clinically confirmed infection was diagnosed in 54.4% (*n* = 110) of the study patients, and a suspected infection was present in 45.6% (*n* = 92). In the septic subgroup, surgical intervention was performed in 11.4% (*n* = 23) of the patients, while 32.7% (*n* = 66) needed mechanical ventilation, 13.4% (*n* = 27) were treated with vasopressors or inotropic agents, and 7.9% (*n* = 16) underwent dialysis. Of the patients with sepsis, septic shock occurred in 12.9% (*n* = 26), and acute renal failure occurred in 47.5% (*n* = 96). Mortality after 28 days was observed in 13.4% (*n* = 27) of the patients with sepsis and 3.9% (*n* = 21) of the patients with other diagnoses. Of the septic patients, 0.5% (*n* = 1) died on day 0, 3.0% (*n* = 6) died on day 1, and 0.5% (*n* = 1) died on day 2.

### 3.3. Diagnostic Performance of Biomarkers

Of all PCT values, 25.1% (*n* = 186) were above the reference value of 0.05 µg/L, whereas this was true for 76.7% (*n* = 559) of the CRP values (cut-off: 5 mg/L) and 31.7% (*n* = 223) of the lactate values (cut-off: 20 mg/dl).

The median PCT value was 0.13 µg/L (IQR: 0.07–0.50 µg/L) and PCT was significantly higher in the patients with sepsis (1.17 µg/L; IQR: 0.25–5.10 µg/L) than in those without sepsis (0.10 µg/L; IQR: 0.06–0.20 µg/L; *p* < 0.0001) (Supplementary Figure S2). The median PCT value in patients who presented with prevalent sepsis at the ED (*n* = 154) was 1.47 µg/L (IQR: 0.39–6.39 µg/L). In patients who became septic within 24 h of admission (*n* = 29), the median PCT value was 0.49 µg/L (0.16–2.24 µg/L), while PCT was lower in patients who developed sepsis more than 24 h after admission to the ED (*n* = 17; median PCT: 0.14 µg/L; IQR: 0.10–2.50 µg/L; *p* < 0.001) (Supplementary Figure S3).

**Figure 1:**
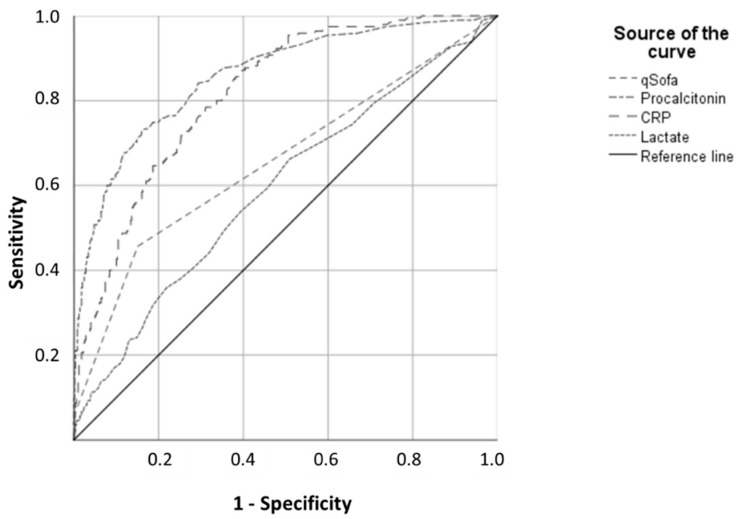
ROC of qSOFA and laboratory parameters for the prediction of sepsis within 96 h of admission. the optimized cut-off value according to ROC-analysis for PCT is 0.26 µG/L. Abbreviations: CRP—C-reactive protein; PCT—procalcitonin. qSOFA—the quick Sequential Organ Failure Assessment.

The AUROC for the PCT value at admission for the diagnosis of sepsis within 96 h was 0.86 (95% CI: 0.83–0.89; *p* < 0.0001). The AUROC for sepsis diagnosis within the first 24 h was 0.87 (95% CI: 0.84–0.90), which was slightly higher than that for the diagnosis of sepsis on day 1 (0.82; 95% CI: 0.78–0.86) and higher still than that for diagnosis on day 2 (0.82; 0.77–0.86 (*p* < 0.0001 for all)). Compared with the other biomarkers, PCT showed higher AUROC values than CRP (AUROC: 0.82; 95% CI: 0.78–0.85; *p* < 0.0001) and lactate (AUROC: 0.60; 95% CI: 0.55–0.64; *p* < 0.0001) (Figure 1). The combination of PCT and qSOFA resulted in an AUROC of 0.86 (95% CI: 0.83–0.89; *p* < 0.00001), while the combination of qSOFA, PCT, and CRP showed an AUROC of 0.88 (95% CI: 0.85–0.01; *p* < 0.00001).

### 3.4. Diagnostic Performance of PCT

The diagnostic performance measures of PCT at various cut-off values for the diagnosis of sepsis are detailed in Table 2. The highest accuracy level of 82.2% correctly classified patients was observed at a cut-off value at 0.50 µg/L. This value resulted in sensitivity of 63.4% (95% CI: 56.3–70.0), specificity of 89.2% (95% CI: 86.3–91.7), a PPV of 68.8% (95% CI: 62.9–74.2), and an NPV of 86.7% (95%Cl: 84.4–88.7).

Further details regarding the diagnostic performance of PCT in the qSOFA subgroups are provided in Table 3.

### 3.5. Logistic Regression Analysis

The logistic regression analyses revealed that at all investigated cut-off values, PCT was a significant predictor of sepsis within 96 h of admission in univariate and all adjusted logistic regression models (Table 4).

### 3.6. Classification Tree Analysis

In the classification tree analysis, PCT and qSOFA were investigated as independent variables to predict sepsis within the first 96 h of admission. The most accurate discrimination was achieved by PCT in the first place at cut-off values of 0.13 and 0.50 µg/L. The most accurate split value for qSOFA in all subgroups was between 1 (first category) and 2 (second category: qSOFA 2 and 3). When qSOFA was forced to be the first variable in the model, the same PCT cut-off values were identified to further discriminate among patients in the qSOFA 1 and qSOFA 2 and 3 subgroups (Supplementary Figure S4). Supplementary Figure S5A,B shows the validation of the decision tree analysis by the split half method.

### 3.7. Net Reclassification Improvement

The NRI of qSOFA by PCT was 22.9% when the established risk categories were applied (qSOFA: 2 points; PCT: 0.50 µg/L; Supplementary Table S3). The NRI increased to 39.9% when the optimised cut-off from classification tree analysis was applied (PCT: 0.13 µg/L and 0.50 µg/L; Supplementary Table S4).

### 3.8. PCT and Mortality

**Figure 2:**
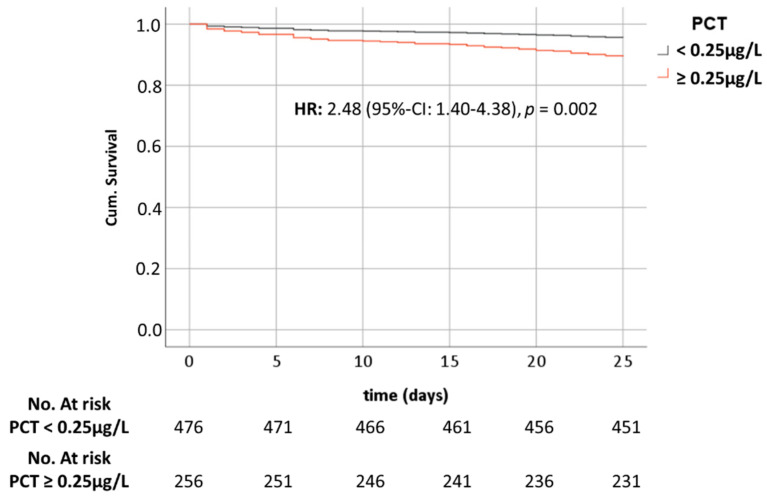
Cumulative survival within 28 days of admission to the ED for patients with procalcitonin (PCT) below, at, or above 0.25 µg/L. Abbreviations: HR—Hazard Ratio. CI- confidence intervalX; Cum: cumulative.

Regarding patients who died within 28 days of admission (6.5%; *n* = 48), the median PCT value was 0.31 µg/L (IQR: 0.12–0.95 µg/L), which was significantly higher than that for those who survived the first 28 days after ED presentation (median: 0.13; IQR: 0.07–0.47; *p* = 0.001). Mortality was significantly higher in patients with a PCT value of at least 0.25 µg/L (10.5%, *n* = 27) than in those with a PCT value below 0.25 µg/L (4.4%, *n* = 21; *p* = 0.001). In the Cox regression analysis, the crude hazard ratio (HR) for PCT at a cut-off value of 0.25 µg/L was 2.48 (95% CI: 1.40–4.38; *p* = 0.002; Figure 2). After adjustment for qSOFA (HR: 1.31; 95% CI: 0.79–2.17; *p* = 0.293), age (HR: 1.04; 95% CI: 1.02–1.07; *p* = 0.001), and gender (HR: 1.66; 95% CI: 0.89–3.09; *p* = 0.114), the HR decreased slightly to 2.13 but was still significant (95% CI: 1.18–3.86; *p* = 0.013). When adjusted for CRP (HR: 1.00; 95% CI: 1.00–1.00; *p* = 0.276) and lactate (HR: 1.03; 95% CI: 1.01–1.04; *p* = 0.003), the HR for PCT was 2.05 (95% CI: 1.03–4.06; *p* = 0.041).

## 4. Discussion

### 4.1. Summary of Findings

To the best of our knowledge, this study is the first prospective study to investigate the diagnostic performance of PCT for sepsis and to employ the qSOFA as entry to the study criteria to focus on a patient population with presumed organ dysfunction rather than infection suspicion alone, as in similar studies to date. This study performed analyses for all qSOFA-elevated subgroups and included the qSOFA 1 population for two reasons. Initially we were seeking patients likely to develop sepsis in addition to already septic patients. Based on our clinical observations, a significant number of patients with qSOFA 1 will deteriorate to qSOFA 2 if not treated promptly. Therefore, qSOFA scores require re-evaluation in EDs. Our results justified our decision, since 19.6% (*n* = 112) of the qSOFA 1 population developed sepsis within 96 h.

Secondly, in the validation cohort of the original study of Seymour et al., among non-ICU encounters the subgroup of qSOFA 1 reported as intermediate-risk encounters was the largest and presented a high mortality rate. In our study, the qSOFA 1 population was also the largest; thus, it was a crucial ED population to include in the analysis.

PCT alone provided an exceptional AUROC of 0.86 (95% CI: 0.83–0.89; *p* < 0.0001), with a slight improvement when combined with a qSOFA score of ≥2 for sepsis prediction. Discriminatory analyses highlighted the diagnostic abilities of PCT on top of qSOFA, proving that PCT acts independently of other established risk markers like CRP and lactate, as revealed in the logistic regression analyses. Furthermore, PCT was an independent predictor of 28-day mortality and was therefore identified as a risk predictor marker.

### 4.2. Clinical Endpoints

In all 742 patients analysed in this study, 202 were diagnosed with sepsis. This high prevalence of sepsis was a result of our selection criteria and accurate assessment of qSOFA score. There were no missing data regarding qSOFA, and the parameters were repeatedly re-evaluated.

In the present study, most of the septic patients (44.6%) were diagnosed with a respiratory infection. Urogenital infection was the second most common source of sepsis in our study population, exceeding abdominal infection; this result contradicted previous studies, including the Impress Study [32], which demonstrated the abdomen to be the second most frequent source of sepsis in Western Europe. A simple explanation for this deviation is that our hospital is the only hospital with a urology clinic in central Berlin and the only one with a kidney transplant unit within a 190-km area.

Furthermore, in opposition of previous studies [33] our results revealed that the age factor showed no statistical significance between the sepsis and non-sepsis groups. The reason for this discrepancy is that we studied a prospectively identified population with elevated qSOFA, namely, patients with presumed organ dysfunction. This led to a homogenisation of critically ill patients of older age with a high proportion of comorbidities in the selected study population, and thus, age did not show a significant association with the occurrence of sepsis in our study. In Germany, the in-hospital mortality of patients diagnosed with sepsis, according to the former definition, was estimated in 2013 to be approximately 41.7% in ICU-treated patients [34]. More recent evidence on mortality regarding community-acquired sepsis using the Sepsis-3 definition is not available. In our study, the 28-day mortality rate was low at only 6.6% since we excluded patients with an already low survival rate (<28 days) attributed to cancer or other conditions, as well as patients with a Glasgow Coma Scale score of below 13 who had no legal representation.

### 4.3. Diagnostic Performance of Biomarkers and qSOFA

Our results regarding qSOFA were consistent with those of previous studies; however, we must acknowledge that the present patient population was an already evaluated qSOFA population. Since the introduction of the qSOFA, numerous studies have attempted to test the performance of the assessment in the screening, diagnosis, and prognosis of sepsis, yielding a range of results from a variety of heterogeneous study populations and leading to many subsequent systematic reviews. In a meta-analysis of 23 studies of patients with infections outside the ICU, Song et al. [35] revealed that a positive qSOFA score had high specificity in predicting in-hospital mortality and sepsis severity but somewhat low sensitivity, making the qSOFA unsuitable as a screening tool. In our study, we confirmed a low sensitivity of 44.6% in predicting sepsis but a high specificity of 85.2%, as expected. However, these results are hardly comparable since the patients in the present study were selected based on their qSOFA scores at admission to the ED. Furthermore, most of the patients in the present study (77.1%) still presented with a qSOFA score of 1 at admission, and further diagnostic measures, including such biomarkers as PCT, would be required to identify the still considerably high proportion of sepsis patients (approximately 20% in the current study) in this intermediate-risk group.

The role of PCT in sepsis, not only in the ICU but also in the ED, has been widely explored in previous studies, revealing contradictory results. In a population with elevated qSOFA scores, our data revealed the notably high diagnostic performance of PCT. Moreover, the AUROC in the present study was similar to the AUROC of the systematic review conducted by Miechun Tan et al. [36], who reviewed nine studies evaluating the diagnostic accuracy of PCT.

Although the low sensitivity of the qSOFA and the high diagnostic performance of PCT were similar to the results of several previous studies, most of those studies were retrospective and investigated the qSOFA score and PCT for sepsis mortality prediction and prognosis [18,19,25]. The results of the present study were consistent, indicating an undeniable benefit from the combination of PCT and qSOFA compared with qSOFA alone for the prediction of sepsis severity and mortality.

In the current study, PCT was investigated prospectively in EDs by a single early measurement promptly after admission, and the main focus was early sepsis diagnosis. PCT exhibited high and independent diagnostic performance at several cut-off values derived from the literature. To achieve the highest possible level of accuracy and thus the optimal trade-off between sensitivity and specificity, a cut-off value of 0.50 µg/L proved to be the optimal value in both the general study population and the qSOFA subgroups. This finding was confirmed through decision tree analysis when PCT was added as a numeric value, and decision tree analysis also revealed another optimised cut-off value of 0.13 µg/L, which could aid in the further identification of low-risk groups. NRI analysis confirmed that PCT was indeed of incremental diagnostic value and improved classification by 23.9% at a cut-off value of 0.50 µ/L. When both cut-off values were applied, NRI improved to 39.9%. The improvement of NRI was mainly triggered by the upgrading of patients with sepsis to higher risk categories, which further illustrates the great clinical benefit PCT can have for early sepsis diagnosis in the ED. Given that the qSOFA score is easily obtainable at admission to an ED, PCT measurement could be recommended for ED patients with a qSOFA score of at least 1 upon admission for further risk stratification. Therefore, the availability of point-of-care PCT measurement in EDs can facilitate the early identification of already septic patients and patients likely to develop sepsis.

### 4.4. Strengths and Limitations

In the present prospective study, no patients had to be excluded for missing values; therefore, we had an advantage over previous studies. On a daily 8-h basis, we screened all patients (approximately 34,000) that presented in the study EDs based on their qSOFA scores.

For the diagnosis of sepsis, the Sepsis-3 definition was used and adjudicated by a panel of experts. For ethical reasons, patients with dementia or a severely altered cognitive state, who had no legally authorised representation, were excluded from the study. This exclusion could be considered as a limitation of our study and may have contributed to the fact that in the qSOFA assessments mental status changes were the smallest contributor to the qSOFA score. In addition, this may have caused a minor reduction in the number of patients with a qSOFA score of 3. The prediction of mortality through PCT should be investigated in further studies since mortality in the present study was low and the subgroup analyses of patients with and without sepsis, as well as full adjustment for all possible other predictors and confounders, was not possible.

## 5. Conclusions

In a cohort of ED patients selected based on current guideline-recommended clinical criteria, PCT exhibited excellent diagnostic performance. PCT can improve early sepsis identification in EDs by 40% (NRI), especially for the majority of patients presenting with qSOFA scores of at least 1 upon admission. Thus, PCT can support clinicians in the early application of targeted measures to improve clinical courses and outcomes. PCT can thus serve as an ideal biomarker for point-of-care measurement in EDs.

## Figures and Tables

**Table 1:** Demographic and clinical characteristics the Charlson Comorbidity Index and qSOFA at admission.

	Total *n* = 742	Sepsis *n* = 202 *	Non-Sepsis *n* = 539 *	*p*-Value
Women % (*n*) **	42.0 (312)	39.6 (80)	42.9 (231)	0.424
Age (median, IQR) **	68 (56–78)	70 (59–78)	67 (56–77)	0.086
Comorbidities % (*n*) **	85.8 (637)	91.6 (185)	3.7 (451)	0.006
Charlson Index points (median, IQR) **	2 (1–3)	3 (1–4)	2 (1–3)	<0.0001
qSOFA items **				
GCS < 15% (*n*) **	9.8 (73)	20.8 (42)	5.8 (31)	<0.0001
Tachypnoea (RR ≥ 22/min) % (*n*) **	71.7 (532)	72.8 (147)	71.2 (384)	0.231
Systolic BP ≤ 100 mmHg % (*n*) **	43.4 (322)	57.4 (116)	38.2 (206)	<0.0001
qSOFA points % (*n*) **				<0.0001
1	77.1 (572)	55.4 (112)	85.2 (459)	
2	20.9 (155)	38.1 (77)	14.5 (78)	
3	2.0 (15)	6.4 (13)	0.4 (2)	
GCS (median, Range) **	15 (3–15)	15 (3–15)	15 (3–15)	<0.0001
BP (mmHg) (median, IQR) **	112 (95–136)	99 (89–122)	117 (96–138)	<0.0001
RR (breaths/min) (median, IQR) **	23 (20–26)	24 (20–26)	23 (20–26)	0.028
Immunosuppresion % (*n*)	10.9 (80)	22.2 (44)	6.7 (36)	<0.0001
WBC/nL (median, IQR) ***	10.0 (7.3–14.4)	12.7 (7.6–17.9)	9.5 (7.2–13.1)	<0.0001
CRP (mg/dL) (median, IQR) ****	34.8 (5.9–99.6)	104.4 (50.0–229.7)	18.3 (3.4–60.9)	<0.0001
Non-survivors day 28% (*n*) *****	6.6% (48)	13.4% (27)	3.9 (21)	<0.0001

Abbreviations: BP—blood pressure; GCS—Glasgow Coma Scale; IQR—Inter Quartile Range; qSOFA—quick sequential organ failure assessment; RR—respiratory rate; WBC—white blood cell count; CRP—C reactive protein * one patient was lost to follow-up ** nmiss = 0 *** nmiss = 11, **** nmiss = 13, ***** nmiss = 10.

**Table 2:** Diagnostic performance of PCT at different cut-off values (recommended by the manufacturer) for the diagnosis of sepsis within 96 h of admission.

	PCT 0.20 µg/L	PCT 0.25 µg/L	PCT 0.50 µg/L	PCT 2.00 µg/L	PCT 5.00 µg/L	PCT 10.00 µg/L
True negative	400	429	481	523	533	537
False negative	44	50	74	122	151	169
False positive	139	110	58	16	6	2
True positive	158	152	128	80	51	33
Sensitivity (95% CI)	78.2 (71.9–83.7)	75.3 (68.7–81.4)	63.4 (56.3–70.0)	39.6 (32.8–46.7)	25.3 (19.4–31.8)	16.3 (11.5–22.2)
Specificity (95% CI)	74.2 (70.3–77.9)	79.6 (75.9–82.9)	89.2 (86.3–91.7)	97.0 (95.2–98.3)	98.9 (97.6–99.6)	99.6 (98.7–100.0)
Positive Likelihood Ratio (95% CI)	3.0 (2.6–3.6)	3.7 (3.1–4.4)	5.9 (4.5–7.7)	13.3 (8.0–22.3)	22.7 (9.9–52.0)	44.0 (10.7–181.8)
Negative Likelihood Ratio (95% CI)	0.3 (0.2–0.4)	0.3 (0.2–0.4)	0.4 (0.3–0.5)	0.6 (0.6–0.7)	0.8 (0.7–0.8)	0.8 (0.8–0.9)
Positive Predictive Value (95% CI)	53.2 (49.2–57.2)	58.0 (53.5–62.4)	68.8 (62.9–74.2)	83.3 (75.0–89.3)	89.5 (78.8–95.1)	94.3 (80.0- 98.6)
Negative Predictive Value (95% CI)	90.1 (87.5–92.2)	89.6 (87.1–91.6)	86.7 (84.4–88.7)	81.1 (79.3–82.8)	77.9 (76.5–79.3)	76.1 (74.9–77.2)
Accuracy (95% CI)	75.3 (72.3–78.4)	78.4 (75.3–81.3)	82.2 (79.2–84.9)	81.4 (78.4–84.1)	78.8 (75.6–81.7)	76.9 (76.9–79.9)

one patient was lost to follow-up. CI—confidence interval.

**Table 3:** Diagnostic performance of qSOFA at a cut-off value of 2 and PCT at different cut-off values within qSOFA categories for the diagnosis of sepsis within 96 h of admission. 95%-CI, 95% confidence intervals.

	qSOFA Cut-Off Score ≥ 2	qSOFA		qSOFA	
		Score = 1		Score ≥ 2	
		PCT Cut-Off	PCT Cut-Off	PCT Cut-Off	PCT Cut-Off
		0.25 µg/L	0.50 µg/L	0.25 µg/L	0.50 µg/L
True negative	459	374	415	55	66
False negative	112	31	42	19	32
False positive	80	85	44	25	14
True positive	90	81	70	71	58
Sensitivity	44.6	72.3	62.5	78.9	64.4
(95% CI)	(37.6–51.7)	(63.1–80.4)	(52.8–71.5)	(69.0–86.8)	(53.7–74.3)
Specificity	85.2	81.5	90.4	68.8	82.5
(95% CI)	(81.9–88.0)	(77.6–84.9)	(87.4–93.0)	(57.4–78.7)	(72.4–90.1)
Positive Likelihood Ratio	3	3.9	6.5	2.5	3.7
(95% CI)	(2.3–3.9)	(3.1–4.9)	(4.8–8.9)	(1.8–3.6)	(2.2–6.1)
Negative Likelihood Ratio	0.7	0.3	0.4	0.3	0.4
(95% CI)	(0.6–0.7)	(0.3–0.5)	(0.3–0.5)	(0.2–0.5)	(0.3–0.6)
Positive Predictive Value	52.9	48.8	61.4	74	80.6
(95% CI)	(46.6–59.2)	(43.3–54.4)	(53.7–68.6)	(66.9–80.0)	(71.5–87.2)
Negative Predictive Value	80.4	92.4	90.8	74.3	67.4
(95% CI)	(78.3–82.3)	(89.9–94.2)	(88.6–92.6)	(65.4–81.6)	(60.6–73.5)
Accuracy	74.1	79.7	84.9	74.1	72.9
(95% CI)	(70.8–77.2)	(76.2–82.9)	(81.7–87.7)	(66.9–80.5)	65.6–79.5

one patient was lost to follow-up. CI—confidence interval.

**Table 4:** Results of the logistic regression analysis of PCT at different cut-off values for the prediction of sepsis in univariate analyses (crude ORs) and adjusted for qSOFA (model 1), adjusted for qSOFA, gender and age (model 2), and adjusted for qSOFA, gender, age, C-reactive protein, lactate, WBC, pre-existing conditions and tumour diseases, IMMUNOSUPRESSION (model 3). One patient was lost to follow-UP.

PCT Cut-Off		OR (Crude)	*p*-Value	OR Adjusted Model 1	*p*-Value	OR Adjusted Model 2	*p*-Value	OR Adjusted Model 3	*p*-Value
0.20 µg/L	Value	10.3	<0.0001	8.7	<0.0001	8.6	<0.0001	4.3	<0.0001
	(95% CI)	(7.0–15.2)		(5.8–12.9)		(5.8–12.8)		(2.6–7.1)	
0.25 µg/L	Value	11.9	<0.0001	10.1	<0.0001	10.0	<0.0001	5.4	<0.0001
	(95% CI)	(8.1–17.4)		(6.8–15.0)		(6.8–14.8)		(3.3–8.8)	
0.50 µg/L	Value	14.4	<0.0001	13.1	<0.0001	13.3	<0.0001	7.7	<0.0001
	(95% CI)	(9.7–21.3)		(8.7–19.8)		(8.8–20.1)		(4.6–13.0)	
2.00 µg/L	Value	21.4	<0.0001	19.4	<0.0001	21.5	<0.0001	12.7	<0.0001
	(95% CI)	(12.1–38.0)		(10.7–34.9)		(11.7–39.4)		(6.2–26.3)	
5.00 µg/L	Value	30.0	<0.0001	28.5	<0.0001	29.8	<0.0001	26.4	<0.0001
	(95% CI)	(12.6–71.3)		(11.8–69.0)		(12.3–72.3)		(8.5–81.7)	
10.00 µg/L	Value	52.5	<0.0001	44.7	<0.0001	47.0	<0.0001	25.0	<0.0001
	(95% CI)	(12.5–220.8)		(10.4–191.8)		(10.9–202.8)		(5.5–114.1)	

OR: odds ratio, CI—confidence interval.

## Data Availability

The data sets used and/or analysed during the present study are available from the corresponding author upon reasonable request.

## Supplementary Materials

The following are available online at https://www.mdpi.com/article/10.3390/jcm10173869/s1, Figure S1: Consort Flow Diagram, Figure S2: Logarithmic illustration of the distribution of PCT in patients with and without a diagnosis of sepsis within the first 96 h after admission. Figure S3: Procalcitonin values stratified by time of sepsis (logarithmic scale). Figure S4: Classification tree containing the independent variables PCT (numeric) and qSOFA (3 categories) as independent variables and the diagnosis of sepsis within 96 h after admission as dependent variable. Figure S5A: Results of the classification tree analysis in the training sample. Figure S5B: Results of the classification tree analysis in the test sample. Table S1: Comorbidities, Table S2: Association of qSOFA criteria and infect foci in all study participants, Table S3. Specification of change of risk categories of qSOFA by PCT applying two established risk groups and resulting Net Reclassification Improvement. Table S4. Specification of change of risk categories of qSOFA by PCT applying three risk groups and resulting Net Reclassification Improvement.
