# Supplementary material for: Diagnostic Performance of Procalcitonin for the Early Identification of Sepsis in Patients with Elevated qSOFA Score at Emergency Admission
Source: J Clin Med. 2021 Aug 28;10(17):3869. doi: 10.3390/jcm10173869 (PMC8432218; doi:10.3390/jcm10173869)

Table S1. Comorbidities.

	Total<br>*n* = 742	Sepsis<br>*n* = 202 *	Non-sepsis<br>*n* = 539 *	*p*-value
Previous myocardial infarction % (*n*)	15.9 (99)	13.4 (23)	16.9 (76)	0.279
Heart failure % (*n*)	37.9 (232)	32.9 (53)	39.3 (179)	0.148
Peripheral arterial disease % (*n*)	12.9 (79)	13.0 (20)	12.9 (59)	0.988
Cerebrovascular dieses % (*n*)	12.8 (92)	15.8 (31)	11.7 (61)	0.140
Dementia % (*n*)	5.4 (39)	4.2 (22)	8.5 (17)	0.020
Chronic respiratory disease % (*n*)	36.5 (257)	29.2 (56)	39.3 (201)	0.013
Collagenosis % (*n*)	2.8 (20)	2.5 (5)	2.8 (15)	0.806
Ulcera % (*n*)	6.2 (39)	6.0 (10)	6.3 (29)	0.891
Mild liver disease % (*n*)	2.8 (20)	6.2 (12)	1.6 (8)	0.001
Severe liver disease % (*n*)	2.1 (15)	3.5 (7)	1.5 (8)	0.088
Hemiplegia % (*n*)	2.6 (19)	4.0 (8)	2.0 (11)	0.143
Diabetes mellitus without end organ damage % (*n*)	20.2 (147)	25.0 (50)	18.4 (97)	0.048
Diabetes mellitus with end organ damage	5.4 (38)	5.7 (11)	5.2 (27)	0.790
Medium to severe renal disease % (*n*)	6.4 (47)	8.5 (17)	5.6 (30)	0.154
Tumor % (*n*)	10.9 (75)	13.2 (25)	10.1 (50)	0.248
Metastatic solid tumor % (*n*)	7.2 (50)	13.6 (26)	4.5 (23)	<0.0001
Leukemia % (*n*)	1.4 (10)	2.5 (5)	0.9 (5)	0.099
Lymphoma % (*n*)	3.2 (23)	6.0 (12)	2.1 (11)	0.007
AIDS % (*n*)	0.5 (4)	1.5 (3)	0.2 (1)	0.032

*one patient was lost to follow up.

Table S2: Association of qSOFA criteria and infect foci in all study participants.

	Pulmonary (*n* = 280)	Urogenital (*n* = 66)	Abdominal (*n* = 58)	Skin or wounds (*n* = 28)	Other (*n* = 16)	Cardiovas cular (*n* = 6)	Central nervous system (*n* = 2)	Unknown (*n* = 55)
**qSOFA points**								
1 % (*n*)	76.1 (213)	59.1 (39)	75.9 (44)	78.6 (22)	81.3 (13)	50.0 (3)	0.0 (0)	72.7 (40)
2 % (*n*)	22.9 (64)	31.8 (21)	22.4 (13)	21.4 (6)	12.5 (2)	50.0 (3)	100.0 (2)	21.8 (12)
3 % (*n*)	1.1 (3)	9.1 (6)	1.7 (1)	0.0 (0)	6.3 (1)	0.0 (0)	0.0 (0)	5.5 (3)
**GCS <15** % (*n*)	8.9 (25)	28.8 (19)	6.9 (4)	10.7 (3)	12.5 (2)	0.0 (0)	100.0 (2)	16.4 (9)
**Tachypnoea (RR ≥ 22/min)** % (*n*)	85.0 (238)	65.2 (43)	53.4 (31)	50.0 (14)	43.8 (7)	100.0 (6)	50.0 (1)	67.3 ()37
**Systolic BP ≤100mmHg** % (*n*)	31.1 (87)	56.1 (37)	65.5 (38)	60.7 (17)	68.8 (11)	50.0 (3)	50.0 (1)	49.1 (27)

Qsofa: the quick sequential organ failure assessment; GCS: Glasgow Coma Scale

Table S3. Specification of change of risk categories of qSOFA by PCT applying two established risk groups and resulting Net Reclassification Improvement.

**Model without PCT**	**Model with PCT at a cut-off value of 0.50 μg/L**		
	risk category 1	risk category 2	
	PCT < 0.50 μg/L	PCT ≥ 0.50 μg/L	
**Patients with sepsis**			**SUM**
risk category 1 (qSOFA = 1)	42	70	112
risk category 2 (qSOFA ≥ 2)	32	58	90
**SUM**	74	128	202
**Patients without sepsis**			
risk category 1 (qSOFA = 1)	415	44	459
risk category 2 (qSOFA ≥ 2)	66	14	80
**SUM**	481	58	539
**Net reclassification improvement**			**22.9%**

Table S3 shows the calculation table for net reclassification improvement when applying two risk categories for both parameters: qSOFA (cut-off value of 2 points), PCT (cut-off value of 0.50 μg/L). PCT: procalcitonin

Table S4. Specification of change of risk categories of qSOFA by PCT applying three risk groups and resulting Net Reclassification Improvement.

**Model without PCT**	**Model with PCT at a cut-off value of 0.13 and 0.50 μg/L**			
	risk category 1	risk category 2	risk category 3	
	PCT < 0.13 μg/L	PCT 0.13-0.50 μg/L	PCT ≥ 0.50 μg/L	
**Patients with sepsis**				**SUM**
risk category 1 (qSOFA = 1)	16	26	70	112
risk category 2 (qSOFA = 2)	7	21	49	77
risk category 3 (qSOFA = 3)	1	3	9	13
**SUM**	24	50	128	202
**Patients without sepsis**				
risk category 1 (qSOFA = 1)	294	121	44	459
risk category 2 (qSOFA = 2)	35	29	14	78
risk category 3 (qSOFA = 3)	1	1	0	2
**SUM**	330	151	58	539
**Net reclassification improvement**				**39.9%**

Table S4 shows the calculation table for net reclassification improvement when applying three risk categories for both parameters: qSOFA points, and the PCT cut-off values of 0.13 μg/L and 0.50 μg/L which were derived from classification tree analysis.

Figure S1. Consort Flow Diagram.

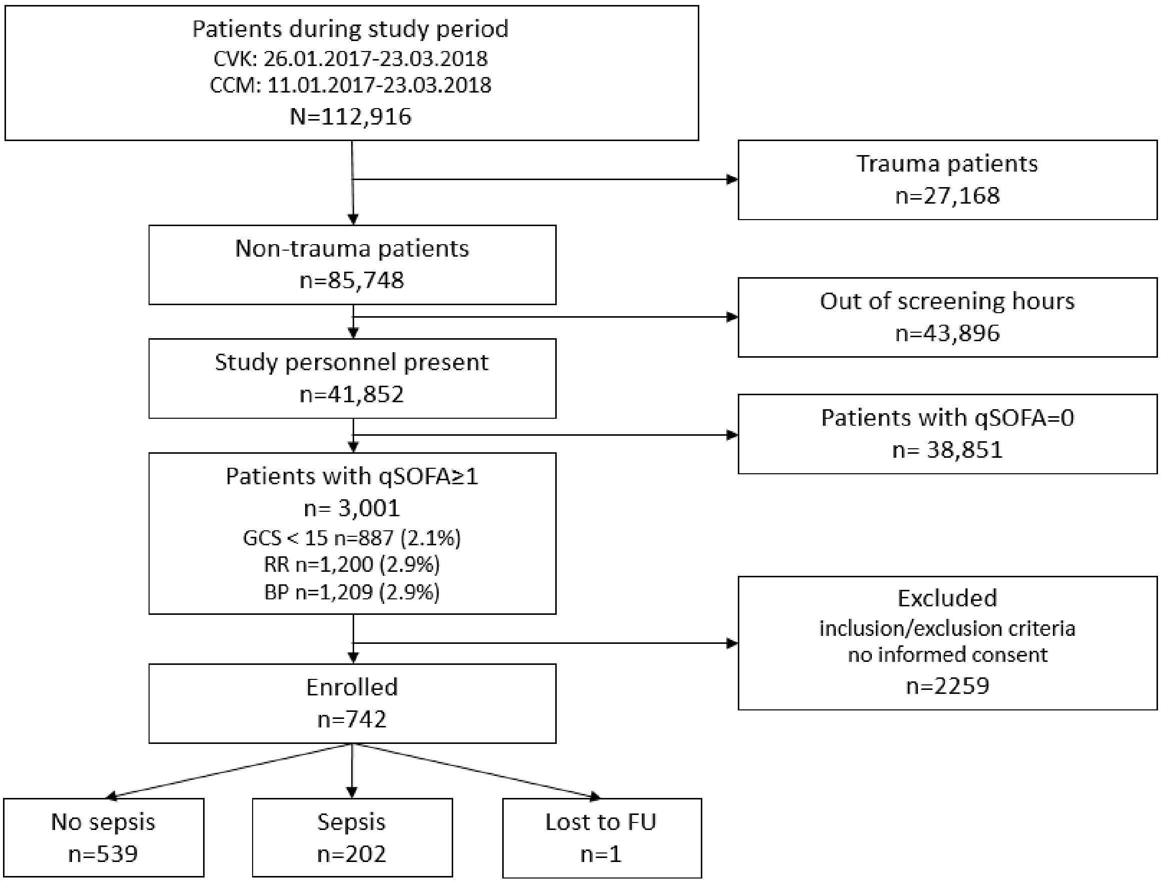

Figure S2. Logarithmic illustration of the distribution of PCT in patients with and without a diagnosis of sepsis within the first 96 hours after admission.

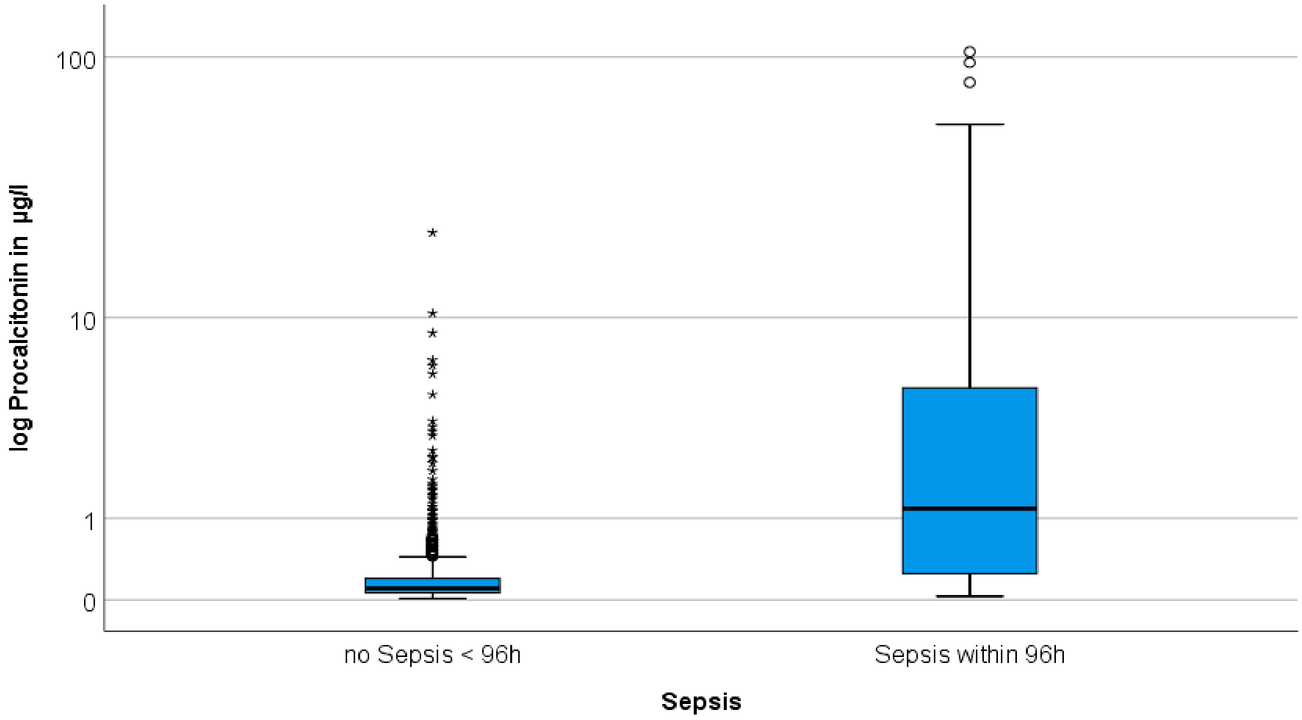

Figure S3. Procalcitonin values stratified by time of sepsis (logarithmic scale).

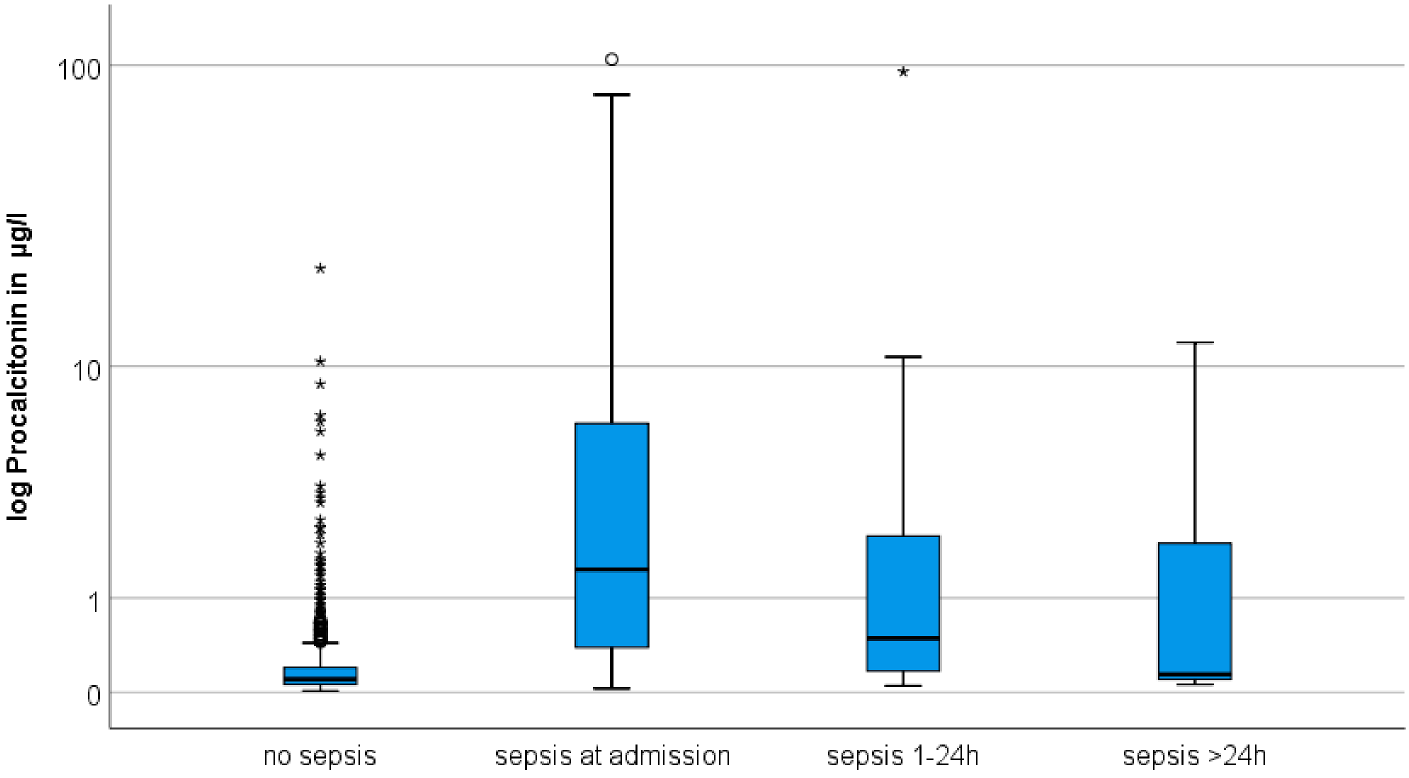

Figure S4. Classification tree containing the independent variables PCT (numeric) and qSOFA (3 categories) as independent variables and the diagnosis of sepsis within 96 hours after admission as dependent variable.

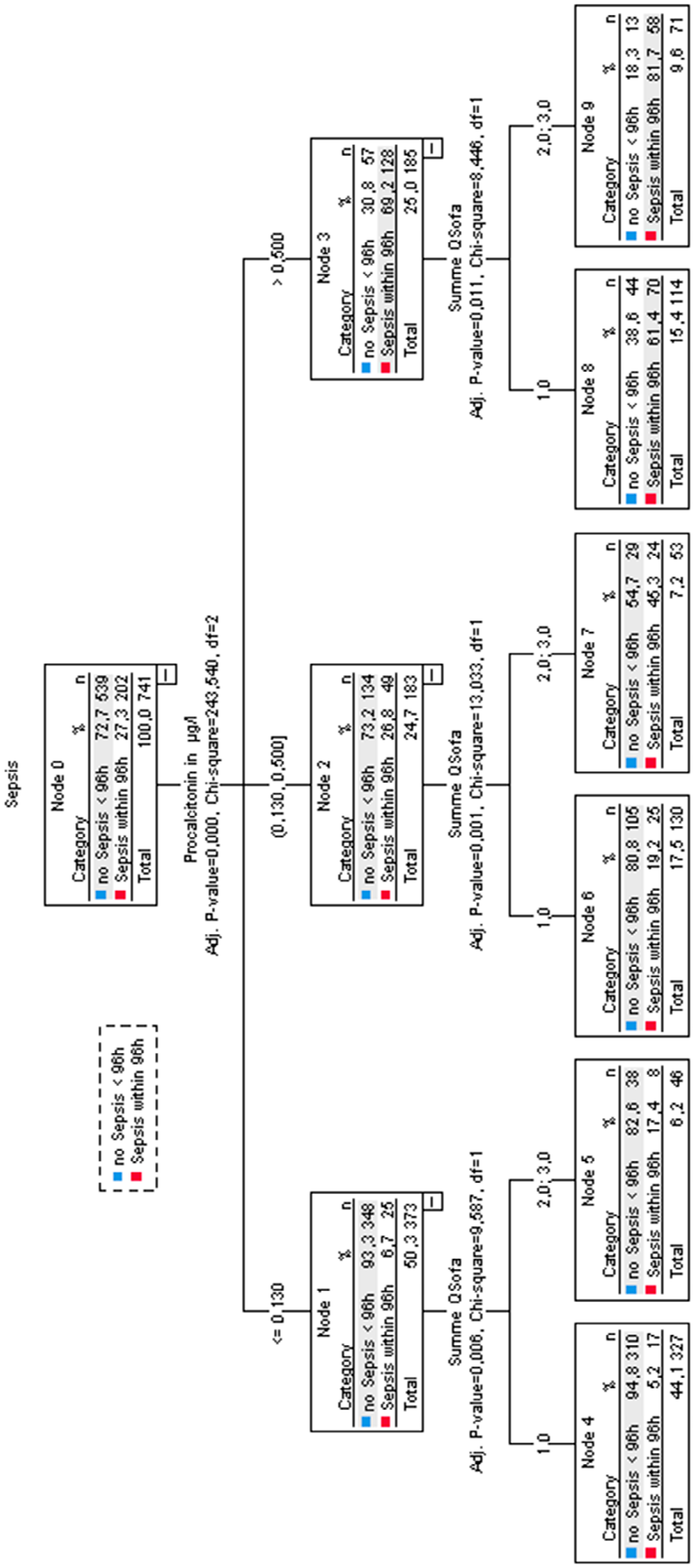

Figure S5. Validation of the classification tree analysis by the split half method. Figure S5A shows the results in the training sample and Supplement Figure 5B in the validation sample of the original data set.

Figure S5A: Results of the classification tree analysis in the training sample.

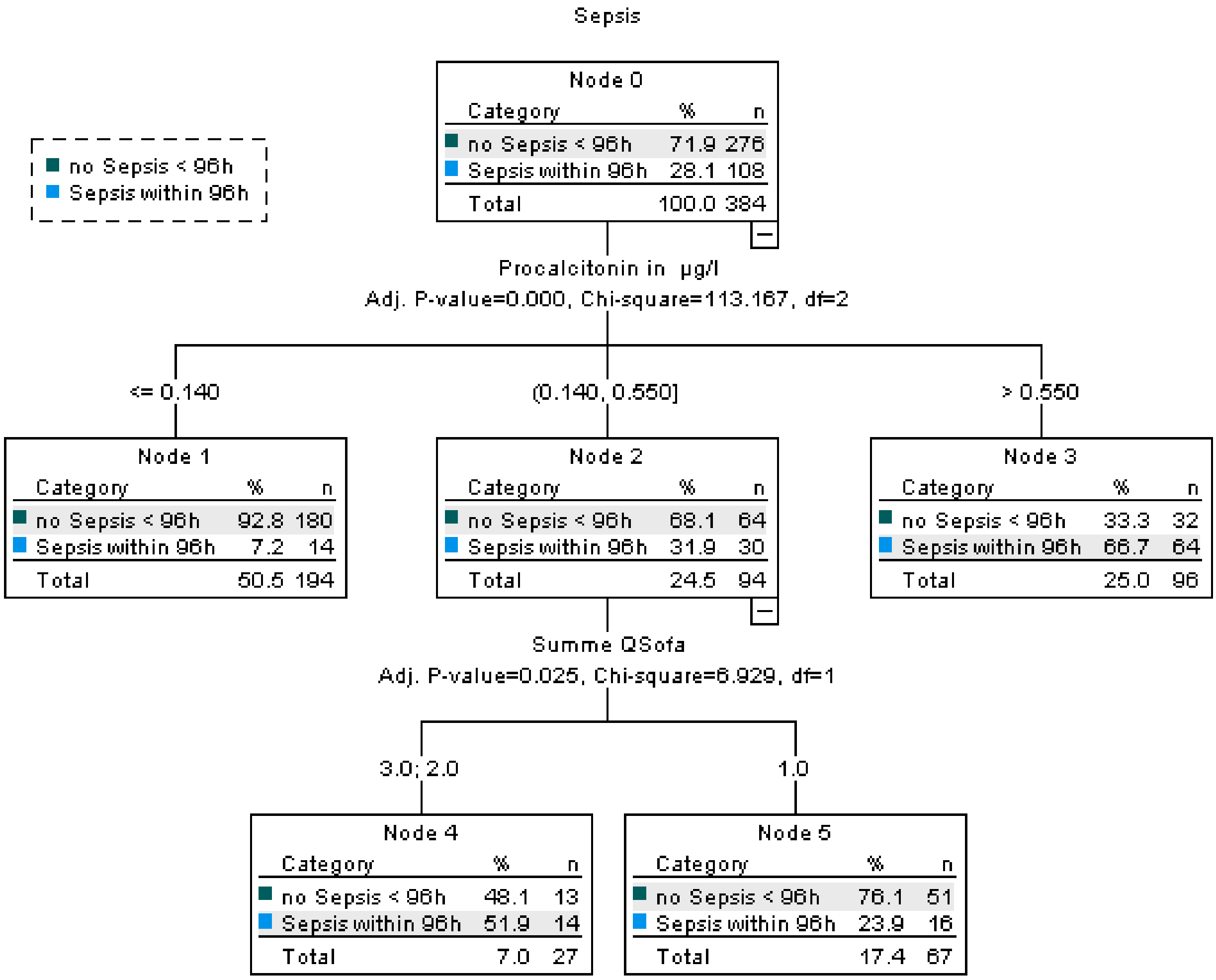

Figure S5B: Results of the classification tree analysis in the test sample.

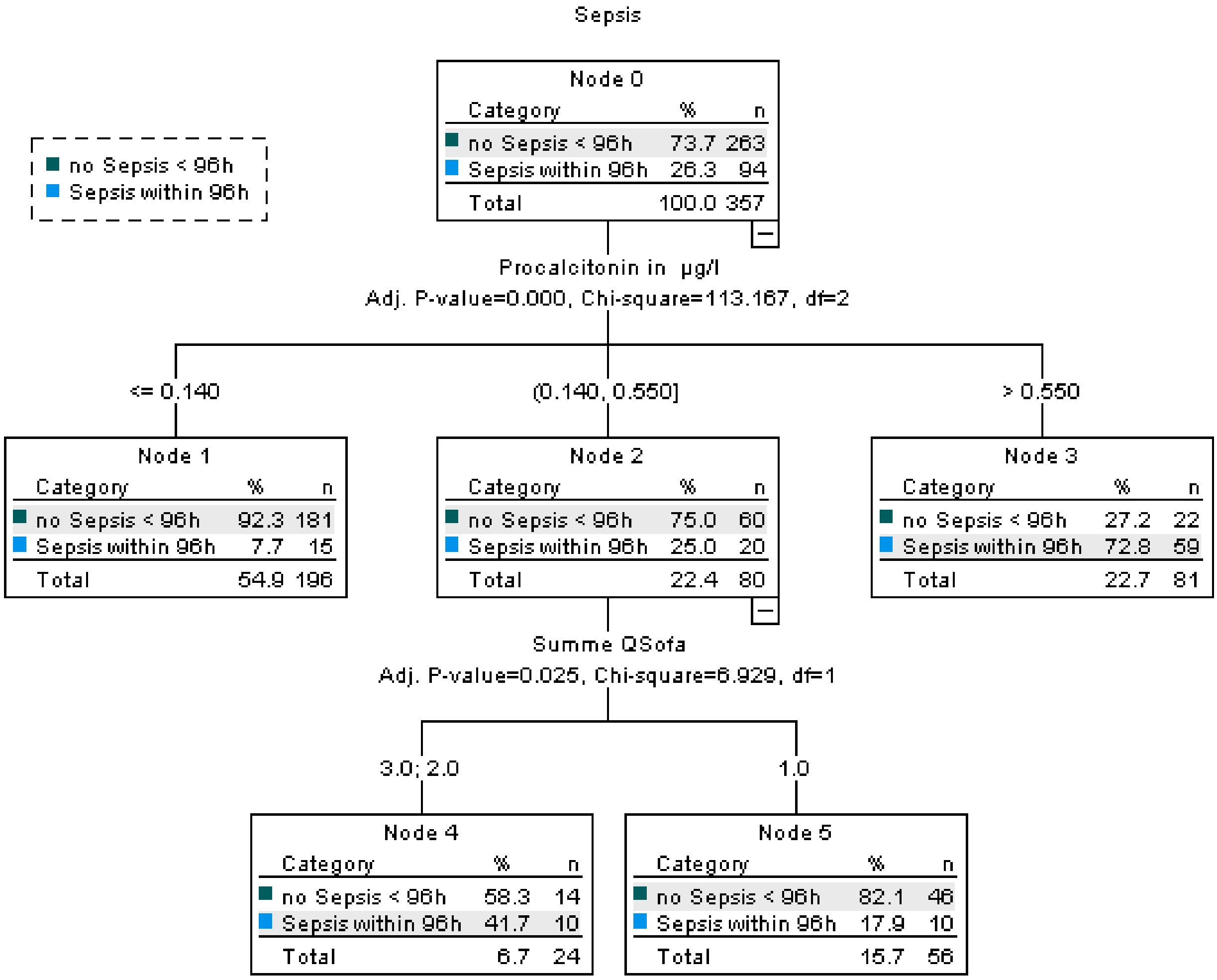

Supplement: Supplementary file 1 [file jcm-10-03869-s001.zip › jcm-1339965-supplementary.pdf]
